# Factors contributing to the immunogenicity of meningococcal conjugate vaccines

**DOI:** 10.1080/21645515.2016.1153206

**Published:** 2016-03-02

**Authors:** Michael Bröker, Francesco Berti, Paolo Costantino

**Affiliations:** a GSK Vaccines GmbH, Marburg, Germany; b GSK Vaccines, Siena, Italy

**Keywords:** carrier protein, glycoprotein conjugate vaccine, immunogenicity, invasive meningococcal disease, polysaccharide, vaccine formulation

## Abstract

Various glycoprotein conjugate vaccines have been developed for the prevention of invasive meningococcal disease, having significant advantages over pure polysaccharide vaccines.

One of the most important features of the conjugate vaccines is the induction of a T-cell dependent immune response, which enables both the induction of immune memory and a booster response after repeated immunization. The nature of the carrier protein to which the polysaccharides are chemically linked, is often regarded as the main component of the vaccine in determining its immunogenicity. However, other factors can have a significant impact on the vaccine's profile. In this review, we explore the physico-chemical properties of meningococcal conjugate vaccines, which can significantly contribute to the vaccine's immunogenicity. We demonstrate that the carrier is not the sole determining factor of the vaccine's profile, but, moreover, that the conjugate vaccine's immunogenicity is the result of multiple physico-chemical structures and characteristics.

## Introduction

The meningococcus *Neisseria meningitidis* (Nm) was identified as one of the causative agents of bacterial meningitis by Weichselbaum in 1887. Reports of this disease date back to 1807 and nowadays meningococci are known as one of the major causes of bacterial meningitis. The incidence of invasive meningococcal disease (IMD) varies according to geographical location and time. Indeed, on occasions a rapid increase from low endemic incidences to epidemic rates occurs. Before the 1920s, without appropriate treatment, IMD was fatal in up to 70% of cases.^1^ Serum therapy, which was recognized and used as a treatment procedure since the early 20^th^ century, improved the outcome and lowered mortality. Following the introduction of antibiotics, the mortality rate decreased to about 10%, but significant long-term sequelae affected approximately 20% of survivors. As in other bacterial species, there is a wide variability of capsular antigens, which were originally serologically defined. This antigenic diversity of meningococcal capsular polysaccharides led to serogroup definition, from which 13 different serogroups have been reported. However, the structure of the serogroup D specific polysaccharide has not been described yet and genetic analyses revealed that the alleged serogroup D isolate was found to contain serogroup C capsule biosynthesis genes with internal stop codons in some genes resulting in an unencapsulated phenotype. Thus, serogroup D does not exist^2^ and the Nm species is mostly limited to 12 serogroups. From a medical perspective, the most important serogroups are A, B, C, W and Y, however, recently, serogroup X has become important in some regions.

The meningococcal polysaccharide capsule antigens are immunogenic in humans and can induce the production of serogroup-specific protective antibodies. Meningococcal polysaccharide vaccines, which have been used for over 40 years, are effective and have proven acceptable tolerability and safety profiles. Nevertheless, plain polysaccharide vaccines have some limitations: as T-cell independent antigens, they act by crosslinking B cell receptors on the surface of naïve B cells to direct plasma cell production; they cannot induce immune memory; they are poorly immunogenic in individuals younger than 2 years of age and may induce hyporesponsiveness. The improved immunogenicity of polysaccharides when conjugated to proteins was originally demonstrated in the 1930s, and the first commercially available conjugate vaccine, *Haemophilus influenzae* type b (Hib) conjugate, which was developed in the 1980s, showed the potential of the conjugate technology.^3^ Thereafter, conjugate vaccines have been developed to protect against meningococcal serogroups A, C and ACWY and are now in broad use.^4^

In conjugate development, the native polysaccharide chain is often slightly downsized to facilitate the conjugation procedures, however, where a more pronounced chain length reduction has been used; then, this antigen is termed an “oligosaccharide.” Although, according to the *International Union of Pure and Applied Chemistry*, the term oligosaccharide is used for a chain length of 3 to 10 sugar residues, the term is used colloquially to describe a significantly shorter version of a polysaccharide (fewer sugar residues and lower molecular weight).

Theoretically, any protein carrying T-cell helper epitopes can be used as a carrier protein for a conjugate vaccine for human use. Traditionally, vaccine manufacturers have used proteins known to have a strong track record of safety and which can be easily produced to provide a high yield at low cost. Two such proteins are tetanus toxoid (TT) and diphtheria toxoid (DT), both chemically-inactivated toxins, which have been used for decades as vaccine components, where toxin inactivation is achieved with substances for example formaldehyde or glutaraldehyde. Two other proteins are also used as carriers for conjugate vaccines: the outer membrane protein complex (OMPC) from Nm (used by Merck for Hib conjugate); and Cross Reacting Material 197 (CRM_197_), a non-toxic mutant of diphtheria toxin, with only one amino acid exchange (at position 52), which results in an enzymatically-inactive toxin, with retained antigenicity. At the beginning of the 1990s, 4 conjugate vaccines were licensed to protect against Hib; they used TT, DT, OMPC and CRM_197_ as carriers.^5^ Additional proteins have been subsequently tested in the development of conjugate vaccines. One such protein, protein D (PD) of non-typeable *Haemophilus influenzae*, is used by GSK Vaccines for 8 out of the 10 polysaccharides in its licensed 10-valent pneumococcal conjugate vaccine (*Synflorix*^™^). Different proteins have been used as carriers not only for the development of Hib and pneumococcal conjugate vaccines, but also for various meningococcal conjugate vaccines.

CRM_197_ does not need chemical agent treatment to inactivate its toxin activity, because it is already genetically inactivated.^6^ The tertiary structure and biochemical and immunological characteristics of CRM_197_ are similar to DT,^7^ and its crystal structure has recently been described.^8^ Using such a mutant protein can have advantages over chemically-inactivated toxins (toxoids), in terms of potentially incomplete detoxification, residual chemical inactivators, control of degree of oligomerization, manufacturing consistency and characterization of the resulting conjugates.^9^

**Figure 1.**
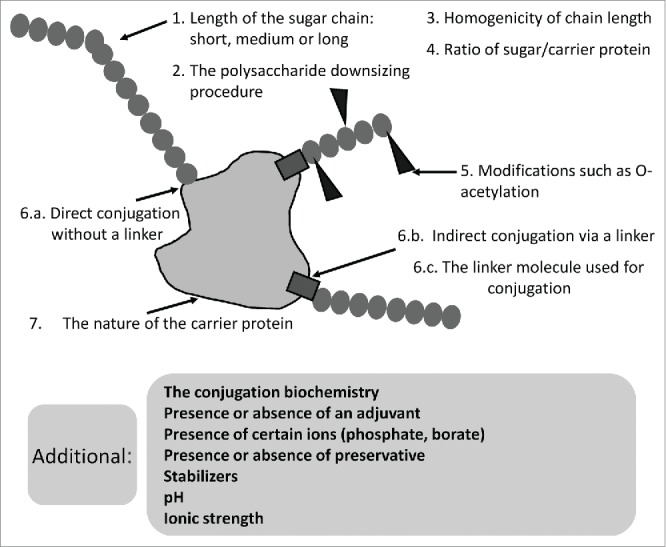
Elements that can have an impact on the immunogenicity of meningococcal glycoconjugates.

Although the carrier protein is of utmost importance in converting the T-cell independent polysaccharide antigen to a T-cell dependent antigen, other parameters, for example saccharide chain length, the linker used for conjugation, and random or selective carbohydrate modification, can have an impact on the immunogenicity of conjugate vaccines. Although publications comparing the immunogenicity of various meningococcal conjugates with different protein carriers, often mention these factors, the focus is predominantly on the carrier as the main factor having an influence on vaccine immunogenicity. Over time, these other aspects of conjugate vaccine formulation have unfortunately been commonly undervalued when characterizing the profile of meningococcal vaccines. This review has therefore been undertaken to highlight these additional properties of meningococcal conjugates and to show that although the carrier protein has an important impact on a vaccine's profile, other factors, schematically shown in Figure 1, can greatly influence the immunogenicity of a meningococcal conjugate vaccine.

Indeed, rather than just considering the carrier, the complex vaccine formulation, with all its specificities, defines a vaccine's overall profile.

## Licensed meningococcal conjugate vaccines

Meningococcal serogroup C (MenC) conjugate vaccines were the first meningococcal conjugate vaccines to be developed and marketed in 1999/2000 and the United Kingdom (UK) was the first country to introduce routine immunization with MenC conjugate in November 1999.

**Figure 2.**
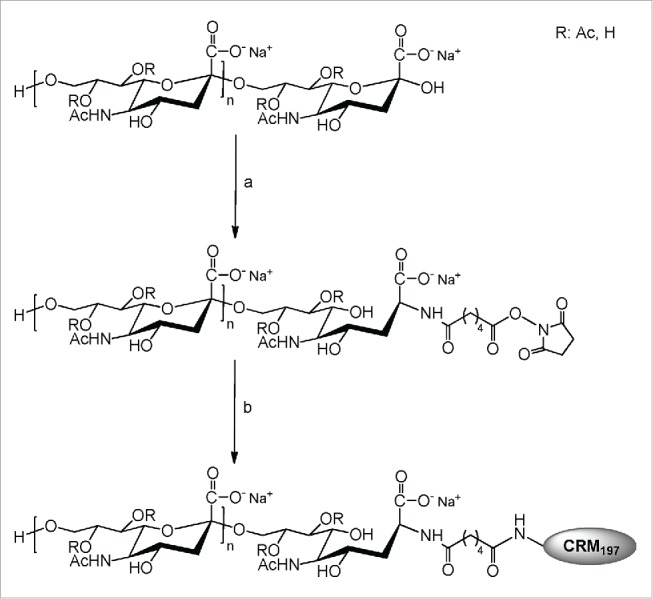
Scheme of the conjugation process used for production of meningococcal serogroup C antigen of MenACWY-CRM conjugate vaccine.

Three MenC conjugate vaccines were licensed at the time: *Meningitec* (Menin-CRM; Nuron Biotech, formerly Pfizer), *Menjugate*^™^ (Menju-CRM; GSK Vaccines, formerly Novartis) and *NeisVac-C* (MenC-TT; Pfizer, formerly Baxter). The first 2 products have CRM_197_ as the carrier and the latter one uses TT. Menin-CRM is prepared by the controlled treatment of the polysaccharide with periodate to generate aldehydes, that are readily conjugated to the amino groups of CRM_197_ by reductive amination. The conjugation chemistry in the manufacture of MenC-TT is similar to Menin-CRM, but the polysaccharide is first de-O-acetylated with sodium hydroxide, followed by limited treatment with sodium periodate and conjugation to TT instead of CRM_197_, by reductive amination. In both cases the periodate treatment causes de-polymerization of the polysaccharides to oligosaccharides, and these are size-fractionated before coupling. The de-polymerization carried out in the production of Menju-CRM is different: the polysaccharide chains are partially hydrolyzed at low pH and size-fractionated before conjugation to CRM_197_ using a bis N-hydroxysuccinimide ester of adipic acid. This conjugation process, as shown for serogroup C in Figure 2, is also used for other capsular serogroups. The immunization program for the introduction of MenC conjugate in England and Wales comprised: a primary immunization schedule at 2, 3, and 4 months of age; a catch-up program of 2 doses for the 5 to 11 month age group and a single injection for individuals aged 1 to 17 years (this was later extended to 24 years of age). This vaccination campaign, using the 3 different MenC conjugate vaccines, was followed by a dramatic reduction in the number of confirmed cases of serogroup C IMD in the immunized age groups in the UK. The observed vaccine effectiveness was 81% overall and 92–97% in teenagers; the numbers of deaths fell from 67 in 1999 to 5 in 2001.^10^ Notably, the MenC-conjugate vaccine (this was later also shown for the MenA-conjugate vaccine) was able to induce herd effect: the protection of unvaccinated individuals in a population, where sufficient people have been vaccinated to thereby interrupt person-to-person bacterial transmission.^11^

Three quadrivalent serogroups ACWY conjugates, *Menactra* (MenACWY-DT; Sanofi Pasteur; to date not licensed in Europe), *Menveo*^™^ (MenACWY-CRM; GSK Vaccines, formerly Novartis) and *Nimenrix* (MenACWY-TT; Pfizer, formerly GSK Vaccines), which use DT, CRM_197_ and TT as carriers, respectively, have been developed and are now internationally marketed. Recently, a monovalent serogroup A conjugate, *MenAfriVac* (MenA-TT; Serum Institute of India Ltd) conjugated to TT, has been developed. It is being distributed in Africa to help protect against IMD, mainly in the ‘meningitis belt’ where there is a high incidence of serogroup A-caused disease. In addition, a bivalent meningococcal serogroup C/Hib combination vaccine, *Menitorix*^™^ (HibMenC-TT; GSK Vaccines) has been developed, which is used mainly in UK and Australia. A three-valent CY/Hib conjugate, *MenHibrix*^™^ (HibMenCY-TT; GSK Vaccines) has also been developed. While all these conjugate vaccines are marketed internationally, some conjugates have been specifically developed for regional distribution (Table 1).

**Table 1.** Overview of licensed meningococcal glycoconjugate vaccines.

Serogroups	Carrier	Trade Name	Manufacturer
Internationally distributed meningococcal conjugate vaccines			
*Monovalent meningococcal conjugates*			
MenC	TT	*NeisVac-C*	Pfizer, former Baxter
MenC	CRM_197_	*Menjugate*^™^	GSK Vaccines, former Novartis
MenC	CRM_197_	*Meningitec*	Nuron Biotech, former Pfizer
*Combined meningococcal conjugates*			
MenACWY	DT	*Menactra*	Sanofi Pasteur
MenACWY	CRM_197_	*Menveo*^™^	GSK Vaccines, former Novartis
MenACWY	TT	*Nimenrix*	Pfizer, former GSK Vaccines
*Combinations with non-meningococcal antigens*			
MenC/Hib	TT	*Menitorix*^™^	GSK Vaccines
MenCY/Hib	TT	*MenHibrix*^™^	GSK Vaccines
Regionally distributed meningococcal conjugate vaccines			
MenA	TT	*MenAfriVac*	Serum Institute of India
MenAC	TT	*MengNa Kang*	Beijing Luzhu (subsidiary of ChongquingZhifei)
MenAC	TT	*Nao Man Ning*	Xiangrui (also known as Beijing Sanroad)
MenAC	TT	*Wo Er Kang*	Yunnan Walvax
MenAC	TT	*MeningACon*	BeijingZhifei Lvzhu Biopharmaceutical
MenAC	TT	N/A	Royal Wuxi

*Note.*

CRM_197_, Cross Reacting Material 197; DT, diphtheria toxoid; Men, meningococcal serogroup; TT, tetanus toxoid

The proteins predominantly used as carriers are TT, DT and the diphtheria mutant toxin CRM_197_. This is not surprising as the toxoids which protect against diphtheria and tetanus have been available for about one century and have demonstrated an acceptable tolerability and safety profile. The vaccine companies have long experience with their production and have sufficient capacities for their use not only as components for diphtheria and tetanus vaccines, but also as carriers for conjugates. Although free, uncoupled CRM_197_ has not been commercially used as a vaccine antigen, this molecule has been intensively characterized and is well defined.^7^

## Meningococcal conjugate vaccines under development

In addition to the currently licensed meningococcal conjugates, other vaccines have been investigated, which interestingly used different carriers to those included in the licensed vaccines.^12-17^

A second MenACWY_TT vaccine is currently in clinical development (*TetraMen-T*; Sanofi Pasteur).^18^ Further, as meningococcus serogroup X is emerging in Africa, various serogroup X conjugates are in development, using either CRM_197_^19^ or TT as carriers.^20^

Following the success of MenA-TT, which has had a profound effect on serogroup A meningococcal infections, a new pentavalent MenACWYX conjugate vaccine is in preclinical development (Research & Development Department, Serum Institute of India, Ltd, Pune, India; PATH, Seattle, Washington, USA). This new thermostable polyvalent meningococcal conjugate vaccine including serogroup X will enter clinical trials in 2015.^21^

## Carrier proteins

### Production and source of carrier proteins

Five carrier proteins are currently used for licensed conjugate vaccines: DT, TT, CRM_197_, non-typeable *Haemophilus influenzae* PD, and the OMPC of serogroup B meningococcus, of which 3 (DT, TT and CRM_197_) are used as carriers for licensed meningococcal conjugate vaccines. DT and TT derive from the respective toxins after chemical detoxification with formaldehyde. CRM_197_, a 58 kDa nontoxic mutant of diphtheria toxin, isolated from the supernatant of *Corynebacterium diphtheriae* C7(β197) tox(−) strain cultures, has the advantage of not requiring chemical detoxification, and is readily purified by a sequence of chromatographic and diafiltration steps.^7^ CRM_197_ can also be recombinantly expressed, e.g., in *Escherichia coli* or *Pseudomonas fluorescens*. Purified recombinant CRM_197_ has been used as carrier for serogroup A conjugate and has been shown to be immunogenic in mice.^22^

The source of the TT used in the production of meningococcal conjugates varies. While Baxter (now Pfizer), which uses TT for the MenC-TT vaccine, procures the carrier from Statens Serum Institut, Copenhagen (Denmark), GSK Vaccines, which uses TT for various conjugate vaccines, obtains TT from its production site in Gödöllö, Hungary. The Serum Institute of India produces its own TT carrier for their MenA-TT. The authors are unaware of any publicly available information on possible differences between the various production methods (e.g., fermentation, down-streaming, toxin inactivation) and subsequent differences in biochemical and immunological features of the toxoids. Regarding CRM_197_ which is used for the production of 2 MenC vaccines, Menin-CRM and Menju-CRM, there is no publicly available information, detailing whether different production and down-stream processes for these 2 diphtheria mutant proteins, employed by the 2 vaccine manufacturers [Pfizer/Nuron Biotech and Novartis (now GSK Vaccines)], result in differences in biochemical characteristics. Recently, Lockyer et al^23^ analyzed the conformation and structure of the TT used as carriers in a variety of glycoconjugates. The authors found that the various TT molecules in these glycoconjugates had different accessibility to carrier-specific monoclonal antibodies, and concluded that the stimulation of B- and T-cells to glycoprotein conjugates and thus the effectiveness of the conjugates may be determined by different carrier structures.

*In vivo* and *in vitro* assays play a vital role in quality control testing of vaccines. When TT and DT are used in glycoconjugate vaccine manufacture, they are required to meet the World Health Organization (WHO) and pharmacopoeial requirements established for the corresponding ‘stand-alone’ vaccine. Additional quality requirements may be considered. Depending on the manufacturing process, DT preparation can show different degrees of purity. Typically, the antigenic purity for DT, as determined by the flocculation test (*in vitro* assay in quality control), should be at least 1500 Lf (limit of flocculation) units/mg protein. Diphtheria toxin is characterized by the presence of dimeric and multimeric aggregation forms, which are also present in the corresponding detoxified preparations. These can be measured using SEC-HPLC (size-exclusion chromatography-high performance liquid chromatography) coupled with a static light scattering detector.^24^

The antigenic purity of TT is typically determined by the flocculation test and should be at least 1500 Lf units/mg of protein. The detoxification process for TT results in oligomerization, the extent of which depends on process conditions. As with DT, the content of monomeric vs. dimeric forms and other aggregates can be determined using methods such as SEC-HPLC coupled with static light scattering detection.^25^

The purity of CRM_197_ batches is expected to be ≥90 %, and often >95 %, as determined by HPLC, sodium dodecyl sulfate-polyacrylamide gel electrophoresis (SDS-PAGE) or capillary electrophoresis. CRM_197_ contains an exposed loop of 3 arginine residues that is clipped by proteases present in the culture medium, resulting in a ‘nicked form’. The intact polypeptide and the 2 fragments A and B, derived by proteolytic clipping followed by dithiothreitol reduction, can be easily detected by SDS-PAGE in reducing conditions. The manufacturing process is expected to produce CRM_197_ with a consistently low degree of nicking.

### Carrier protein amount

The amount of carrier proteins used in the various monovalent meningococcal vaccines can differ; it can also differ among the different conjugates in combination vaccines. However, it is usually between 5 and 35 µg.

According to the summary of product characteristics (SmPC) for the 2 MenC_CRM_197_ conjugates, the amount of CRM_197_ is in a similar range: 15 µg for Menju-CRM and 10–20 µg for Menin-CRM. The total amount of CRM_197_ used for MenACWY-CRM is about 47 µg (32.7 to 64.1 µg). The amount of CRM_197_ to which the 4 oligosaccharides are individually linked varies: serogroup A: 12.5 to 33 µg; serogroup C: 6.25 to 12.5 µg; serogroup W: 3.3 to 10 µg; serogroup Y: 3.3 to 10 µg. The MenACWY-DT conjugate contains 48 µg of DT.

Although different amounts of TT have been clinically analyzed in various formulations (22.1, 34.0, 37.1 and 44.0 µg),^26^ the total amount of TT in MenACWY-TT is 44 µg. There is no detailed information in the SmPC about the quantities of TT used for each of the 4 oligosaccharides. However, it should be noted at this point that not only the nature of the carrier, but also the amount of a given carrier used for a distinct conjugate can have an impact. The amount of TT in the MenA-TT conjugate is 10–33 µg compared with 10–20 µg for the MenC-TT conjugate. In the HibMenC-TT combination, the Hib-specific polysaccharide PRP (polyribosyl-ribitol phosphate) is conjugated to 12.5 µg TT, while the meningococcal serogroup C polysaccharide is conjugated to 5 µg. In the 3-valent HibMenCY-TT conjugate, the TT concentrations are: 5 µg for the serogroup C polysaccharide; 6.5 µg for the serogoup Y polysaccharide; 6.25 µg for PRP.

Clinical studies with conjugates using different amounts of TT (and therefore different polysaccharide to protein ratios) found different immune responses.^26^ The 2 variables: amount of carrier protein and polysaccharide to carrier protein ratio, can have an impact on both the immunogenicity and the immune response to the polysaccharide (and also to the carrier), but concurrent additional variations in the experimental vaccines (for example, by using a spacer or direct conjugation) makes it generally difficult to assess the impact on low or high loading of the carrier with polysaccharide molecules.

### Carrier-specific immunogenicity

Besides imparting T-cell help for the immune response to the attached polysaccharide, the carrier protein can induce a carrier-specific antibody response. Studies from population-based serum analyses in the Netherlands, where MenC-TT has been used in children since around 2002, showed a booster response and higher TT antibody titers in vaccinated children as compared to age groups who had not received the vaccine.^27^ Burrage et al^28^ studied the immune response to diphtheria and tetanus following a MenC conjugate booster in children at school entry (between 3.5 and 6 years) and school leaving (13 to 18 years); the children received a diphtheria-tetanus vaccine either one month before, one month after, or concurrently with one of the 3 MenC conjugates, with either CRM_197_ or TT as carrier. There were no clinically relevant negative interactions identified, and immune responses were observed in almost all children, suggesting that protection was developed against diphtheria, tetanus, and meningococcal antigen. A phase 4 study, carried out in the United States and in Italy, analyzed the carrier protein immune response in adolescents induced by MenACWY-CRM. In the group receiving MenACWY-CRM concomitantly with TdaP (tetanus-diphtheria-acellular pertussis) and HPV (human papillomavirus) vaccines, the DT antibody concentrations were substantially higher compared to the group receiving placebo+TdaP+HPV vaccines; the TT antibody concentrations were similar in both groups, indicating that CRM_197_, as part of the conjugate vaccine, was able to boost the diphtheria immune response.^29^

Overall, clinical studies have demonstrated that significant increases in diphtheria antitoxin levels were generated by MenC-CRM and MenACWY-CRM vaccines, and significant tetanus antitoxin responses were generated by MenC-TT and MenACWY-TT vaccines. However, these vaccines are not licensed for booster immunization to help protect against diphtheria or tetanus.

In a study of adults ≥56 years in Lebanon, Dbaibo et al^30^ measured a significant immune response to TT following MenACWY-TT administration. Before vaccination, 6.3% MenACWY-TT recipients had anti-TT ≥0.1 IU/mL (international unit), which increased to 28.1% post-vaccination. Nevertheless, in the MenACWY-TT SmPC, and also in the SmPCs or package leaflets for the other meningococcal conjugates, it is clearly stated that these conjugate vaccines should not be regarded as immunogens to protect against diphtheria or tetanus.

To date, no clinical studies have addressed whether the carriers, when administered as conjugates to immunological naïve persons, can induce a protective immune response to tetanus or diphtheria. Thus, although it is well-known that the TT, DT and CRM_197_ carriers can induce a significant booster response, it is not yet known if these carriers can induce a protective (a toxin-neutralizing) immune response in man. In order to generate optimal immunogenic responses to CRM_197_ carrier protein, DT priming in infants is required.^31^ Dbaibo et al^30^ concluded that one injection in formerly unprimed individuals may need further booster(s) in order to reach an effective immune response to TT. However, the low response to TT may also be the result of hidden epitopes, due to the specific conjugation process used for this vaccine, or by the immunological characteristics of TT itself, due to the specific production methods for this toxoid.^23^ This is confirmed by results published by Basta et al^32^, who found that single-dose immunization of vaccinees in Mali with MenA-TT did not induce persistent tetanus immunity, which might be explained by the limited exposure of this population to primary tetanus vaccination before MenA-TT vaccination. Recently, Borrow et al^33^ reported that MenA-TT induced consistent tetanus serologic responses in 1- to 29-year-olds in a vaccination campaign in the sub-Saharan Africa countries, comparable to those expected after a booster dose of TT. They also reported a 25% decrease in cases of neonatal tetanus among countries that completed MenA-TT campaigns in persons aged 1–29 years.

In this context, non-clinical protection experiments with various conjugates, carried out in animals according to the European Pharmacopeia (2008), may give some insight into the different immunogenicity potential of the carrier proteins TT, DT and CRM_197_. One dose of meningococcal conjugate vaccine based on TT (HibMenC-TT) was able to protect mice against a lethal challenge with tetanus toxin, and a conjugate vaccine based on DT (MenACWY-DT) was able to protect guinea pigs against a lethal challenge with diphtheria toxin.^34^ However, even 2 doses of conjugate vaccines based on CRM_197_ (MenC-CRM; MenACWY-CRM) failed to protect guinea pigs against a lethal challenge with diphtheria toxin.^34^ It therefore appears that CRM_197_, which differs in one amino acid from diphtheria toxin (and DT), and has not been treated with formaldehyde, has lower immunogenic/protective capacity than DT, when used as carrier protein in meningococcal vaccines and assessed in guinea pigs.

The main vaccines used to prevent diphtheria, tetanus and pertussis are DTwP (diphtheria-tetanus-whole-cell pertussis) or DTaP (diphtheria-tetanus-acellular pertussis) vaccines or broader combinations. Primary vaccination of infants and young children generally consists of 3 doses of either DTwP or DTaP in the first year of life followed by a booster in the second year of life, and further booster vaccinations using Td or a Td combination according to national recommendations. Chinese scientists recently reported that diphtheria and tetanus antibody titers decline more slowly in children who receive DTwP compared to DTaP; and emphasized that booster vaccinations against diphtheria and tetanus should be strengthened.^35^ Such a booster could be achieved using conjugate vaccines based on either DT, CRM_197_ or TT, using the “dual use” or “windfall effect” of these vaccines, whereby an immune response is induced not only to the polysaccharide antigens but also to the carriers. Thus, while in many countries recommended adolescent boosters including D, T, IPV (inactivated poliovirus vaccine) and aP are often delivered using combination vaccines, such as TdaP, Td-IPV or TdaP-IPV, these vaccines can be concomitantly given with meningococcal vaccines including MenC or MenACWY conjugates. If concomitant vaccination is delivered with meningococcal conjugate vaccines, the booster to D or T would be increased by the carrier proteins DT, CRM_197_ or TT component, dependent on which conjugate is used. As reported above, conjugates have useful potential as boosters for previous tetanus/diphtheria immunizations. However, some authors conceived that the optimal carrier in a conjugate vaccine should induce a low level of anti-carrier antibodies.^36^

Two other relevant aspects have been studied: (i) how pre-exposure to the unconjugated carrier (carrier priming), as a result of infant immunization schedules, influences the response to conjugated carbohydrates when these are subsequently administered to children and (ii) the role of the carrier in ‘immunological interference’, that might occur when conjugate vaccines are formulated into larger combinations. Although several preclinical^s1-s9^ and clinical studies^s10-s24^ have been published (see separately listed citations at the end of the references section), it is difficult to draw general conclusions and merits a separate review.

## Conjugation process

The steps involved in the preparation of polysaccharide-carrier conjugates include activation of the polysaccharide and sometimes also of the protein, and the conjugation. The meningococcal conjugate vaccine polysaccharides can be conjugated to the carrier proteins either directly, without a linker molecule, or using a linker (spacer). In general, vaccine producers consistently use a technique either to directly couple the polysaccharide to the carrier protein, or one based on a spacer molecule, which is generally attached to the polysaccharide, although in some cases to the carrier proteins. Very few comparative data have been published. If a spacer is used by the vaccine producers, they generally use the same spacer for different vaccines. Comparative studies into different linkers are limited and to date, only a few spacers have been used to produce meningococcal conjugates; the effect of spacer vs. no spacer has seldom been investigated. However, the spacer can potentially create neoantigenic structures, that can either have no effect or alternatively lead to unwanted non-protective antibodies. This aspect has been investigated in animal models, and it is generally recommended to avoid linkers containing rigid cycles in favor of flexible structures.^37-39^

When polysaccharides are directly linked to the carrier protein, important antigenic epitopes may be sterically shielded by the bulky protein. The use of a linker molecule between the polysaccharide and the carrier protein may decrease this steric shielding and result in the outward presentation of the polysaccharide on the carrier protein to the immune cells; more antigenic epitopes may therefore be available to stimulate and mature antigen presenting cells. Examples of a direct conjugation are MenC-TT and Menin-CRM, where the meningococcal group C polysaccharide is oxidized with sodium periodate and conjugated directly to TT or CRM_197_, respectively. Examples in which a linker is used are Menju-CRM and MenACWY-CRM, both of which include the short di-carboxylic acid, adipic acid (backbone of 6 carbon atoms). In these 2 vaccines, adipic acid connects the end reducing group of the polysaccharide chain to the ϵ-amino group of one of the 39 lysine residues within the CRM_197_ molecule. Due to sterical conformation, the 39 lysine residues are however not equably accessible for conjugation. Because the conjugation chemistry is highly selective and limited in its orientation, this avoids the formation of lattice-like structures and reduces complexity and heterogeneity of the resulting conjugate; the oligosaccharides are arranged radially from the CRM_197_ molecule. The adipic acid di-hydrazide (ADH) is used as the spacer in the MenACWY-DT manufacturing process.^40^

The nature and the length of the spacer can have an impact on the immunogenicity of the conjugate. During the development of a serogroup X conjugate vaccine, Micoli et al^19^ compared the short adipic acid (6 carbon atoms) against a longer 12 carbon atom-based linker. The conjugate with the longer spacer induced higher serum bactericidal antibody (SBA) titers in mice after 2 doses, indicating that the length or nature of the spacer had had an effect on the antibody response.^19^ Huang et al^41^ tested a heterobifunctional polyethylene glycol (PEG) molecule as a spacer arm to conjugate serogroup Y polysaccharide to TT. Using a PEG molecule of 2 kDa significantly increased the immune response in mice and increased the polysaccharide IgG (immunoglobulin G) titers by 3-fold, and the length of immune persistence compared to a direct conjugation of the polysaccharide to the carrier protein. The authors concluded that PEG can decrease the steric shielding effect of TT on antigenic epitopes of the polysaccharide due to its linker chain.

During MenACWY-TT development, the direct conjugation of serogroup C polysaccharide to TT was compared to conjugation using the adipic acid spacer. Results from clinical studies showed that the serogroup C conjugate with the spacer induced higher SBA titers in both children and individuals aged 15–25 years, compared to the conjugate without the spacer; the percentage of toddlers reaching rSBA titers ≥8 was also higher.^26,42^ The post-vaccination immune response against serogroup A polysaccharide was also higher in young adults aged 15–25 years when the vaccine formulation included a spacer, as compared with one using direct conjugation.^42^ Consequently, for further MenACWY-TT vaccine development, an adipic acid linker was used in the production of both the serogroup A and C components, while serogroups W and Y polysaccharides are conjugated directly to TT. In MenACWY-TT, the conjugation process is random, not oriented and results in heterogeneous lattice-like structures.

For polysaccharide activation, there are 2 main method categories: random activation along the chain by periodate oxidation or cyanylation and end-group specific activation. Sodium periodate oxidizes diols (2 adjacent carbons with hydroxyl groups) into aldehydes (RHC=O) and in this process breaks C-C bonds. Thus, depending upon the polysaccharide structure, periodate activation can fragment (downsize) a polysaccharide containing in-chain diols or open the ring structure of monosaccharide repeating units, which can alter the conformation, and potentially also the immunogenicity of the polysaccharide. The cyanylation chemistry using 1-cyano-4-dimethylaminopyridinium tetrafluoroborate (CDAP) allows a direct conjugation (without the use of a spacer) of polysaccharide antigens to proteins. CDAP reacts with the free OH groups of the polysaccharide with no chain breakage, creating cyanoester groups highly reactive with amino groups of proteins to form covalent linkages.

The activation chemistry and the length of the conjugated polysaccharide are generally interconnected and determine the 2 main categories: polysaccharide-protein or oligosaccharide-protein conjugates. For example, methods based on specific activation of terminal groups are typical of fragmented polysaccharides to oligosaccharides, or synthetic ones, in order to increase the concentration of reacting end groups. This is the case for Menju-CRM and MenACWY-CRM, where oligosaccharides obtained by hydrolysis of the homologous polysaccharides, and subsequently activated with a linker, react with the free ϵ-amino group of lysine on the CRM_197_. Other examples are the meningococcal group C conjugates MenC-TT and MenC-CRM, wherein polysaccharide chain diols are oxidized with periodate with simultaneous fragmentation and formation of reactive aldehydes.

In MenA-TT production, the periodate method is used to activate the polysaccharide; only the sugar residues lacking an O-acetyl group on carbon 3 can be activated by periodate treatment.^43^ The percentage of O-acetylation in the purified serogroup A polysaccharide in MenA-TT is 77–85%.^44^ The TT is activated by introducing hydrazide (-NH-NH_2_) groups to the exposed aspartic and glutamic acids carboxyl groups, by reacting with hydrazine, in the presence of a soluble carbodiimide. The activated TT is then incubated with the periodate-activated polysaccharide at pH 6.5 to 7.5; the conjugate mixture is subsequently reduced by sodium borohydride and diafiltrated to exclude small molecules.^45^

## Polysaccharides

### Purification of polysaccharides

Negatively charged meningococcal polysaccharides can be precipitated from the culture filtrate by the addition of quarternary ammonium salts, such as cetavlon, to the bacterial culture supernatant. The polysaccharide can then be purified from the cetavlon complex through a combination of solubilization, selective precipitation, filtration, diafiltration and/or chromatographic steps, depending on specific manufacturers' protocol. While it has been shown that calcium and magnesium counter ions increase the hydrolytic rate of Hib capsular polysaccharide as compared to sodium,^46^ and that adsorption of Hib conjugates onto aluminum hydroxide adjuvant results in the hydrolysis of the polysaccharide and in the liberation of unconjugated oligomers, with possible negative impact on immunogenicity,^47^ little is known about the effect of the type of counter ion and formulation buffer/composition on the properties of meningococcal polysaccharides and corresponding conjugates. Studies on the effect of the counter ion on the conformational properties of serogroup A meningococcal polysaccharide revealed that ammonium counter ion is associated with single chains of elongated conformation, sodium counter ion promotes folding in a sort of globular conformation while the addition of calcium ions favors aggregation of the globular forms to form a toroidal-like structure;^48^ the impact on immunogenicity and stability of these different conformations has not been studied.

While the polysaccharides have been produced so far in the pathogenic bacteria expressing the polysaccharides of interest, Fiebig et al^45^ recently produced serogroup X polysaccharide in *Escherichia coli* by cloning and expression of the capsule polymerase in this host. This approach may offer a safe (non-pathogenic organism) and economic alternative to produce recombinant polysaccharides in vaccine development.

### Length of polysaccharides

The length of polysaccharide chains differs: the antigen of Menju-CRM has about 14–19 repeating units, that of Menin-CRM contains 20–47 repeating units^49^ and MenC-TT is a “fragment.” During MenA-TT development, it was demonstrated that conjugates with a short chain length are less immunogenic in mice compared to longer chains conjugated to TT.^43^ This suggested that the length of the poly/oligosaccharide chains can have an impact on the immunogenicity of meningococal conjugates. The impact of chain length upon Hib conjugate immunity has recently been analyzed by Rana et al,^50^ who showed a greater immunogenicity of low molecular weight PRP_TT conjugates in comparison with their high molecular weight counterparts. It is nevertheless difficult to draw general conclusions without considering the role of conjugation chemistry.

While polysaccharides are commonly prepared from bacterial cultures, Wang et al^51^ synthetically prepared serogroup W polysaccharides of various lengths. The authors prepared oligosaccharide chains consisting of 2, 4, 6, 8 or 10 repeating saccharide residues and coupled these to CRM_197_. Following immunization of mice, Wang et al^51^ analyzed the immune sera and found that the disaccharide conjugate could not induce bactericidal antibodies. Tetrasaccharides were the minimal length to elicit antibodies with bactericidal effects, and the biggest amount of antibodies with bactericidal abilities was induced by octasaccharides.

Liao et al^52^ designed and prepared a series of serogroup C specific α-2,9-oligosialic acids without O-acetylation and of different length, and coupled them with keyhole limpet hemocyanin as the carrier. The order of immunogenicity of the conjugates following immunization in mice was tri>di>tetra>penta, suggesting that larger glycans were not necessarily the better immunogens.

Recently, Morelli et al^53^ prepared conjugates with fully synthetic serogroup X oligomers fused to CRM_197_. Following immunization in mice of the conjugates with aluminum phosphate as adjuvant, it was shown that the conjugated trimer was the minimal length possessing immunogenic activity, despite having significantly lower immunogenic activity than a pentadecamer oligosaccharide version prepared from the native polysaccharide and linked to the same carrier.

These limited results indicate that no clear statement can be made about the impact of polysaccharide antigen length on its immunogenicity. Moreover, the effect of chain length may depend upon the chemistry of the polysaccharide (the serogroup), the carrier protein used and the polysaccharide/protein linkage.

Two generally different methods are used to produce size-reduced polysaccharides (oligosaccharides). The first uses controlled mild acid hydrolysis and size fractionation, whereby the resulting oligosaccharide is then reductively aminated and activated by coupling through amine group to a functional linker, bis-N-hydroxysuccinimide ester adipic acid. The reaction between the activated oligosaccharide and the protein generates the conjugate. The second method produces size-reduced polysaccharides by periodate oxidation generating aldehyde groups. Upon incubation with the carrier protein, there is a formation of Schiff's bases between the amino groups of the protein and the aldehyde groups of the oligosaccharide, which can be reduced to form stable covalent bonds by treating with sodium cyanoborohydride.^49^ Hydrogen peroxide has also been used to generate meningococcal serogroup C oligosaccharides and the reducing end groups derivatized with L-Tyrosine hydrazide to form an hydrazide-derivatized saccharide for characterization studies.^54^

In MenACWY-CRM and Menju-CRM, the polysaccharides are consistently downsized to oligosaccharides under defined mild acid conditions. The fractions with the desired defined chain length (number of sugar residues) are collected and used for the subsequent conjugation process. No data are publicly available concerning the polysaccharide downsizing process used for MenACWY-TT.

### Polysaccharide to protein ratio

The ratio of the polysaccharide to the carrier is a key chemical parameter and can have an impact on immunogenicity, as shown for Hib conjugate vaccine.^55^ For MenACWY-CRM, this ratio is highly consistent from lot-to-lot and is well defined: 0.5–0.6 for the MenA and MenC components; 1.0 for the MenW component; 0.6–0.7 for the MenY component.^56^ The glycosylation is also reflected in the increased molecular masses of the single conjugates, as compared with CRM_197_. Upon downsizing, the antigens are size-fractioned and antigens of a distinct length with a defined molecular mass are collected. The average polysaccharide molecular mass is: serogoup A, 88.5 kDa; serogoup C, 85.2 kDa; serogroup W, 110.1 kDa and serogroup Y, 84.6 kDa.^56^ For other licensed meningococcal conjugates, no information is publicly available. The WHO Technical Report Series on meningococcal conjugates provides guidance on quality control testing at each stage in vaccine manufacture and analysis of the protein/polysaccharide ratio is part of the release criterion to show consistency of the manufacture.^57^

### Amount of polysaccharides in the vaccine

While the 3 licensed monovalent serogroup C conjugates and the MenA-TT conjugate all contain 10 µg polysaccharide, the polysaccharide concentration used in the 3 quadrivalent conjugates differs; in combination vaccines the serogroup C component is less than 10 µg. GSK analyzed different polysaccharide concentrations (2.5 and 5.0 µg) in clinical studies and opted for 5 µg for all 4 polysaccharides in MenACWY-TT.^26^ For MenACWY-CRM, the manufacturer uses 10 µg serogroup A, and 5 µg for the 3 other antigens. For MenACWY-DT, the manufacturer uses 4 µg for the 4 polysaccharides.

McVernon et al^18^ have studied various formulations of a MenACWY-TT conjugate, with different polysaccharide doses. The authors compared the immunogenicity of a single “low” [4 µg each of serogroup A, C, Y and W polysaccharide coupled to either 22.1 µg or 33.9 µg TT], “medium” [10 µg of each polysaccharide coupled to 36.6 µg TT] or “high” dose vaccine [10 µg of each polysaccharide coupled to either 54.8 µg or 84.8 µg carrier protein] in toddlers. All vaccines were immunogenic. However, for serogroup A, 10 µg polysaccharide was more immunogenic than those formulations containing 4 µg. Similar results were obtained for serogroup W and C polysaccharides. Interestingly, the immune response to serogroup C was highest in toddlers who had received a monovalent MenC-TT vaccine containing 10 µg polysaccharide (identical to the highest concentration in the quadrivalent vaccine), as a comparator. Formulations with higher serogroup A and C polysaccharide content were more immunogenic than formulations with lower content. However, no clear differences in immunogenicity were determined for serogroup Y. Recently, the Expanded Program on Immunization of the WHO entitled a 5-µg formulation of MenA-TT for use in infants.^58^

### O-acetylation of polysaccharides

Capsular polysaccharides from many pathogenic bacteria are O-acetylated, and a common feature is that the O-acetyl groups are easily removed at alkaline pH. While serogroup X polysaccharide is not O-acetylated, the polysaccharides of serogroups A, C, W and Y can be. One or more hydroxyl groups in position 4, 7, 8 and 9 can most commonly be substituted by O-acetyl groups. The meningococcal genes coding for this modification of various serogroups have been analyzed.^59^

For some conjugates, the manufacturer de-O-acetylates the naturally O-acetylated polysaccharide and this can be achieved using various conditions. Lee et al (Lee SM, Purification of Group C Meningococcal Polysaccharide for a Conjugate Vaccine. Oral session presented at: 12^th^ Annual World Vaccine Congress; 2012 Apr 10–13; Washington DC, United States) reported that the percentage of remaining impurities (protein and nucleic acid) in the polysaccharide fraction depends upon the de-O-acetylation process. When a mild base treatment is used (0.1 N NaOH at 25°C), the percentage of both protein and nucleic acid is 12% and 7% respectively, however, when a harsh base treatment (>0.1 N NaOH and higher temperature) is used, the percentage of impurities is much lower (0.55% for protein and 0.25% for nucleic acid). Further purification can be carried out using ultrafiltration.

#### Serogroup C

The serogroup C polysaccharide is a homopolymer of α-2→9 linked N-acetylneuraminic acid with O-acetyl groups distributed exclusively between C-7 and C-8 of its sialic residues (OAc+). Freshly isolated serogroup C polysaccharide indicates that on the surface of the organism, most of the O-acetylation exists at position C-8, with some regions containing O-acetylated or de-O-acetylated C-7 sialic acid. After extraction of polysaccharide and storage in solution, most of the O-acetylated groups migrate to C-7, leaving an epitope that is conformationally related, but not identical, due to the presence of the O-acetylated group, to one contained in the de-O-acetylated serogroup C polysaccharide (OAc-). A study carried out in the 1970s in the USA demonstrated that about 15% of serogroup C isolates lack the O-acetyl groups and a subsequent study in the UK at the end of 1990s revealed that about 12% of isolates were OAc-.^60^ Michon et al^61^ produced partially or completely de-O-acetylated serogroup C polysaccharide by treating the antigen with dilute base, and examined the immunogenicity of OAc- vs. OAc+ forms of serogroup C polysaccharide. The immune response in mice was highly dependent on the degree of O-acetylation. Less O-acetylation resulted in higher SBA titers toward the O-acetylated serogroup C strain C11, which was dominant during the late 1990s in the UK. Since an unconjugated OAc- serogroup C polysaccharide was previously shown to be highly immunogenic in humans, North American Vaccine Inc. (later taken over by Baxter) adopted the OAc- form to develop a MenC-TT conjugate.^61^

#### Serogroup A

The serogroup A polysaccharide is a homopolymer of N-acetyl-mannosamine-phosphate linked α-1–6. It is 70 to 90% O-acetylated at C-3. As O-acetylation at C-3 of serogroup A polysaccharide is an important factor in its immunogenicity, de-acetylation during the conjugation process can result in a reduced immune response. Berry et al^62^ demonstrated that in 17/18 post-immunization human sera, the degree of inhibition, measured using enzyme-linked immunosorbent assay (ELISA), indicated that serogroup A specific antibodies mostly identified a polysaccharide antigen with O-acetyl residues involved. Comparative immunogenicity studies in mice with OAc+ and OAc- forms of serogroup A antigen revealed that de-O-acetylation resulted in a marked loss of immunogenicity when the immune response was measured by ELISA. Most importantly, the ability to induce functional bactericidal antibodies was drastically reduced by de-O-acetylation. Nevertheless, epitopes not involving O-acetyl groups may contribute to the development of protective response since mice vaccinated with OAc- antigen did develop some functional antibody.^62^

In a clinical study, 2 formulations of a MenACWY-TT with either 68% or 82% polysaccharide O-acetylation were used to immunize healthy adults aged 18 to 25 years. As there was no significant difference in the percentage of vaccinees achieving rSBA ≥8 and GMTs (geometric mean titers), the authors concluded that the level of O-acetylation did not affect the immunogenicity of the vaccine.^63^

#### Serogroup Y

According to a study from the UK for the period 1996 to 2001, approximately 80% of serogroup Y isolates express O-acetyl groups and about 8% of serogroup W strains (with significant differences between the years).^64^ During the Hajj outbreak^65^ in the associated serogroup W (ST11) complex, all strains were de-O-acetylated.

The O-acetyl moiety is probably present at C-7 of the serogroup Y polysaccharide at the surface of the bacteria; after purification and storage in solution, these O-acetyl groups eventually migrate to the neighboring hydroxyl groups, i.e. C-9. Fusco et al^66^ used serological studies to explore the importance of the O-acetyl groups for the protective immunogenicity of the serogroup Y polysaccharide. Immunogenicity experiments in mice revealed that the OAc- conjugates consistently generated higher polysaccharide specific IgG titers, when using de-O-acetylated polysaccharide conjugated to human serum albumin and coated to ELISA plates, and also induced higher SBA. In another experiment, competitive inhibition bactericidal assays were performed to assess the importance of O-acetyl groups in the binding of functional killing mouse and human serogroup Y specific antibodies to live meningococci using an OAc+ strain. Serogroup Y polysaccharides were used as inhibitors but with different states of O-acetylation: at C-7, at C-9, and OAc-. The inhibition assays were performed with mouse sera raised against OAc- and OAc+ serogroup Y conjugated to TT. The OAc- polysaccharide inhibitor was consistently better (6 to 13-fold) than the OAc+ polysaccharide for both the OAc+ and the OAc- strains. The authors suggested that the O-acetyl groups may mock an important epitope to the immune system and thereby misleading the antibody response resulting in an escape mechanism. The general role of O-acetylation of polysaccharides is yet debated.

#### Serogroup W

In some strains, the sialic acid of the disaccharide repeat of W polysaccharide is O-acetylated at residues C-7 or C-9. The polysaccharide of most clinical isolates is OAc-, but currently licensed serogroup W polysaccharide and conjugates contain the OAc+ form.

Giardini et al^67^ investigated the impact of the OAc+ vs. the OAc- form on serological measurements of anti-W IgG antibodies in immunized adults. Overall, there was no difference in functional activity, as measured by SBA against OAc+ and OAc- W target bacteria. However, their data indicate that for some sera the agreement in anti-OAc+ vs. anti OAc- W IgG assignments was serum-specific and did not reflect the *in vitro* functional (killing) activity. Jin et al^68^ analyzed the immunogenicity of MenW polysaccharide conjugated to *Staphylococcus enterotoxin* C1 having low levels of O-acetylation in mice; removal of these O-acetyl moieties had no adverse effect on the observed immunogenicity.

Gudlavalleti et al^69^ studied the effect of O-acetyl groups on the immunogenicity of W polysaccharide coupled to hydrazine derivatized TT (after periodate oxidation of the polysaccharide) in mice. Using three different variants of the polysaccharide: (i) OAc+ form; (ii) naturally O-acetyl negative form; and (iii) chemically prepared OAc- form, they showed that O-acetylation does not contribute an important epitope in raising bactericidal antibodies. Although the OAc+ form induced some OAc+ specific antibodies, these did not appear to contribute to the bactericidal effect. The authors found higher bactericidal titers for conjugates made using naturally O-acetyl negative and OAc- polysaccharides, compared to OAc+ conjugates both to OAc+ and OAc- target strains. The authors concluded that OAc- may have an advantage as a starting material for the preparation of W polysaccharide conjugate vaccines.

## Adjuvants

In general, adjuvants for human vaccines are used for various purposes: i) to increase the immunogenicity of antigens; ii) to enable a more rapid immune response; iii) to broaden the immune response; iv) to possibly reduce the amount of antigen in a vaccine, without reducing the immune response needed for protection; v) to possibly reduce the number of injections; vi) to selectively stimulate either a Th1 (type 1 T helper cell) or Th2 (type 2 T helper cell)-related immune response; vii) to bind certain components in the vaccines which might be pyrogenic, such as lipopolysaccharides (especially from gram-negative bacteria) or lipooligosaccharides (LOS, e.g., from meningococci) to thereby reduce the amount of free pyrogenic compounds and increase the tolerability; viii) to increase the shelf-life of a vaccine by increasing antigen stability.^70^

All marketed plain meningococcal polysaccharide vaccines, as well as discontinued vaccines are non-adjuvanted. The three licensed MenC conjugate vaccines contain aluminum salts. Menju-CRM and MenC-TT contain aluminum hydroxide (1.0 mg and 0.5 mg Al^3^^+^, respectively), Menin-CRM contains aluminum phosphate (0.125 mg as Al^3^^+^ content) and the monovalent MenA conjugate (MenA-TT) also contains aluminum phosphate. When alum is used as an adjuvant, the binding of the antigen to alum needs to be controlled, because the immunogenicity of an antigen can differ depending on whether it is adsorbed to alum or is non-adsorbed in the vaccine. The three licensed MenC conjugates are stable when stored both at the recommended and increased temperatures^71^ and the presence of aluminum salts may contribute to this effect. Otto et al^72^ analyzed the binding characteristics of individual saccharides and protein components of MenC conjugate vaccines. They found that at neutral pH, binding of the TT-based MenC conjugate to AlPO_4_ was primarily promoted through the carrier protein rather than through the polysaccharide, while CRM-based MenC conjugates considerably bind to Al(OH)_3_ due to electrostatic interactions.

Each manufacturer utilizes propriety material and its own confidential standardized production processes, which differ among the various aluminum salts, but which all fulfill the European and United States Pharmacopeia guidelines. The biophysical and biochemical properties of the different adjuvants differ and the individual aluminum adjuvants used in the various vaccines cannot be exchanged among each other. Trace amounts of elementary impurities in commercially available aluminum hydroxide adjuvants can have an impact on the stability of vaccine antigens. For example, it was recently shown that even low ppm (parts-per-million) concentrations of copper can act as a catalyst, leading to the formation of free sulfite radicals and resulting in the auto-oxidation and degradation of an antigen.^73^

In contrast to the monovalent MenC conjugates, the HibMenC-TT combination vaccine and the 3 conjugated 4-valent MenACWY conjugate vaccines do not contain adjuvants. Thus, there is no general rule, as to whether meningococcal conjugates should be developed with or without adjuvant. Early formulations of MenACWY-CRM contained aluminum phosphate, but clinical studies in which 2 different formulations (with vs. without alum) were compared against plain polysaccharide vaccine in adolescents, showed that both formulations were non-inferior to plain polysaccharide vaccine, but induced higher SBA GMT compared to polysaccharide vaccine. As there were no clinically meaningful differences in the immunogenicity of the adjuvanted and non-adjuvanted conjugates, and following specific regulatory guidance, only the non-adjuvanted formulation was developed.^74^ However, for the MenX-TT vaccine (Serum Institute of India Ltd.), which is still in development, the addition of aluminum phosphate has been shown to significantly improve the immune response in mice, in terms of inducing both specific antibody titers and the functional antibody titers.^20^

While all licensed meningococcal polysaccharide conjugates contain either no adjuvant or aluminum salts as adjuvant, various other adjuvants have been preclinically assessed. The adjuvant MF59 (an oil-in-water emulsion) can significantly enhance the immune response to influenza antigens; it is a component of a seasonal trivalent inactivated egg-based influenza vaccine (*Fluad*; Seqirus, formerly Novartis Vaccines) licensed for adults ≥65 years, and of various pandemic and pre-pandemic influenza vaccines.^75^ Three different formulations of serogroup C conjugate antigen have been tested in infant baboons: phosphate buffered saline (PBS, no adjuvant); aluminum hydroxide or MF59. Animals vaccinated with MF59-containing vaccine produced significantly more specific antibodies and higher bactericidal antibody titers compared to the groups which received vaccine with either no adjuvant or aluminum hydroxide.^76^ Nevertheless, no clinical program has been initiated with a MF59 formulated meningococcal vaccine.

Wang et al^51^ compared the immunogenicity of serogroup W polysaccharide conjugate coupled to CRM_197_ and found that an α-galactosylceramide derivate used as adjuvant (and not licensed for human use) was more immunogenic in mice than a conjugate in which an aluminum salt was used.

Qiao et al^77^ chemically linked β-glucan to TT as a carrier for serogroup Y polysaccharide. β-glucan is a stimulator of humoral and cellular immunity and a serogroup Y_TT_glucan conjugate could increase both the polysaccharide and TT-specific immune response.

Mutants of the *Escherichia coli* enterotoxin (LTK63 and LTR72) and trimethyl chitosan induced a mucosal immune response to MenC conjugates when mice were immunized via the intranasal route.^78,79^ Alternatively, Brynjolfsson et al^80^ observed that the BCG (Bacillus Calmette-Guerin) vaccine enhanced the immune response to MenC conjugate vaccine in mice neonates; while the immune response after MenC conjugate vaccination was preferentially IgG1 antibody type, it was more of a mixed IgG1 and IgG2a/IgG2b antibody response and increased antibody titers with bactericidal activity were measured when BCG vaccine was simultaneously applied.

Although the 2 latest meningococcal serogroup B vaccines are not polysaccharide conjugates, it should be mentioned that *Bexsero*^™^ (GSK Vaccines, formerly Novartis Vaccines) contains aluminum hydroxide as adjuvant and *Trumenba* (Pfizer, formerly Wyeth Vaccines) contains aluminum phosphate

## Excipients

### Buffers

The various meningococcal vaccines contain different solvents: NaCl, phosphate buffer or Tris (tris(hydroxymethyl)-aminomethane); Trometamol) buffer and sometimes additives, such as sucrose or mannitol as stabilizers. The solvents and buffers may principally have an impact on the stability of the vaccine formulations and therefore on their immunogenicity. For example, the buffer can influence the adsorption of MenC-TT antigen to aluminum hydroxide: Experiments using either PBS or saline (150 mM NaCl), showed that when PBS was used, the antigen tended to desorb from the adjuvant, but this did not occur if saline was used.^81^ The authors discussed that desorption of antigen from aluminum hydroxide by phosphate ions had been previously reported and that phosphate anions can adsorb to aluminum hydroxide and lower the isoelectric point of the adjuvant. This in turn may decrease the electrostatic interaction with negatively-charged antigens, such as serogroup C polysaccharide. The authors recommended that phosphate buffer should be avoided in adsorbed vaccine formulations and if buffer capacity is required to maintain the stability of the vaccine, then Tris buffer should be used. According to SmPCs, some meningococcal vaccines including MenA-TT, MenACWY-TT and HibMenC-TT conjugates contain Tris buffer. A high NaCl concentration (3.75–5.75 mg per 0.5 mL) is included in the MenAC_TT conjugate vaccine *Wo Er Kang*, manufactured by Yunnan Walvax, China.

### Preservatives

While all marketed monovalent MenC and quadrivalent MenACWY conjugates do not contain preservatives, the monovalent MenA-TT vaccine contains 0.01 % thiomersal, an organic mercury-based preservative. Thiomersal may induce type IV hypersensitivity reactions and some concerns exist regarding neurotoxicity and the potential to induce some immunological disorders.^82^ This compound can also have an impact on the immunogenicity of vaccine antigens by modulating the Th2 responses.^83^ For example, the presence of thiomersal stimulates poliovirus degradation, with a concomitant decrease in immunogenicity,^84^ and also stimulates the dissociation of foot-and-mouth virions, leading to lower immunogenicity.^85^ For meningococcal conjugate vaccines, no studies have been reported to date, regarding whether the presence of thiomersal has an effect on the stability and/or immunogenicity of the antigens

## Conclusions

It is the formulation of a conjugate as a whole which determines the immunogenicity profile. There are many factors which can have an impact on the potency of a vaccine and these factors have been discussed in this review. The use of a given carrier protein is only one factor, which can have an impact on the immunogenicity of a glycoconjugate; it is not the sole or decisive one. Nevertheless, as documented in the following examples, the clinical relevance of increased or decreased immunity to the polysaccharide antigen, by using a certain carrier protein is unknown:

Monovalent MenC conjugates are available with either CRM_197_ or TT as carrier proteins. Two conjugate vaccines containing CRM_197_ as the carrier protein: Menju-CRM and Menin-CRM, are often termed “CRM-based conjugates,” when being used as a comparator vaccine to assess the immunogenicity of another conjugate, e.g., a TT-based conjugate. However, it is not appropriate to attribute these 2 vaccines based only on their carrier, because there are at least 3 differences in the formulation of these 2 vaccines that may also affect their immunological profile:

1. Menju-CRM contains aluminum hydroxide, while Menin-CRM contains aluminum phosphate;
2. Polysaccharide downsizing in Menin-CRM is performed using periodate treatment, while in Menju-CRM mild acid treatment is used;
3. In Menin-CRM, conjugation is carried out directly to the oligosaccharide without a linker, while in Menju-CRM the reducing ends of the polysaccharide chains are activated and coupling to the carrier is achieved with a linker.

Clinical studies have shown that Menju-CRM is more immunogenic than Menin-CRM. Borrow and Findlow^86^ concluded that the immunogenicity of Menju-CRM is comparable to the TT-based serogroup C vaccine MenC-TT, and that MenC-TT and Menju-CRM, but not Menin-CRM, fulfil sufficient immunogenicity criteria to allow a one-dose immunization strategy in infants (instead of 2 or 3 doses in the first year of life), with a booster in the second year of life for infant vaccination in the UK. Based on a publication by Findlow et al,^87^ in which the immunogenicity of a single dose of MenC-TT or Menju-CRM was evaluated in infants, the Joint Committee on Vaccination and Immunisation^88^ concluded in January 2012 that MenC-TT or Menju-CRM, the serogroup C conjugates currently used in the childhood immunization program in the UK, can be administered to infants as a single dose to provide protection. However, as none of the available serogroup C vaccines are licensed for a single dose in infancy, this would be an “off-label” indication (Menin-CRM has not been evaluated due to its lower immunogenicity). In fact, only these 2 serogroup C conjugates are currently in use in the UK for the one-dose infant vaccination schedule, which became effective June 1, 2013. Thus, it becomes evident that the carrier protein alone cannot be the deciding factor for the overall immunogenicity of Menju-CRM and Menin-CRM. There must be other and/or additional factors which have an impact on the immunogenicity of these 2 conjugates and at least one of the 3 points listed above may contribute to the immunogenicity of these 2 vaccine formulations.

A higher immunogenicity of Menju-CRM compared to Menin-CRM may also be deduced from clinical studies in which the serogroup C component of MenACWY-TT was compared against either Menin-CRM or Menju-CRM. In various studies, the immunogenicity of the MenC component in MenACWY-TT was always shown to be higher than the immune response to Menin-CRM. In a clinical study^89^ in which the immunogenicity of MenACWY-TT was studied in 2 to 10 year-old children, the immune response to the monovalent Menju-CRM vaccine was higher and induced significantly higher rSBA titers (5,291.6 [confidence interval: 3,814.6–7,340.5]) than after MenACWY-TT (2,794.8 [confidence interval: 2,393.5–3,263.3]). In this case, the differences between the 2 vaccines are manifold. The most obvious difference is the carrier protein: TT vs. CRM_197_, but there are several others: While the serogroup C component in MenACWY-TT contains 5 µg polysaccharide, Menju-CRM contains 10 µg; Menju-CRM is adjuvanted with aluminum salt while MenACWY-TT is non-adjuvanted; there could be interference of the other 3 serogroup polysaccharides in MenACWY-TT which may result in lower immune response to serogroup C polysaccharide. These observations highlight that premature conclusions, deducing that the difference in immunogenicity of 2 compared vaccines is due to different carrier proteins, should be avoided. The reasons for different immunogenicity may be caused by other factors, and in this case the impact of differences in polysaccharide concentration, the presence or absence of adjuvant and/or to possible interference by other antigens, is not known. Other less obvious reasons may also be important.

The three aforementioned production procedures for Menin-CRM and Menju-CRM can contribute to the overall immunogenicity profiles. It is not known how much each of the different procedures contributes to the immunogenicity of the resulting conjugate, but it is obvious that the carrier protein cannot be the only and decisive factor, because CRM_197_ is used in both vaccines.

MenACWY-DT only has a modest immunogenicity in infants.^90^ There was no significant immunologic advantage conferred by increasing the dosage beyond 4 µg for each serogroup antigen.^90^ The perception among many experts and physicians is that the low immunogenicity of this vaccine in infants is due to carrier DT, which was used to develop this quadrivalent vaccine. Other factors, such as the conjugation process, sugar/protein ratio, length of the polysaccharide, have not been considered as having an impact on the immunogenicity of the vaccine. Thus, only the preparation of a MenACWY conjugate, in which only the carrier is replaced by another protein, but all other components and the production process are unchanged, could determine whether it really is the carrier protein which is responsible for the relatively low immunogenicity of this vaccine in infants.

Conjugate vaccines are semi-synthetic biologicals; they have complex structures and differ from each other by many factors. The use of a certain protein as carrier is just one of the several factors that will impact on the overall potency of the vaccine.

The immunogenicity of a conjugate is determined by its complex formulation and production process; immunogenicity can not be due to only one factor.

Each meningococcal conjugate has to be carefully analyzed in clinical studies, to identify its potential to induce the production of bactericidal antibodies and help protect against IMD. Conjugates cannot be regarded as generics and even if 2 conjugates seem to be similar, their immunogenicity can differ due to one or more factors discussed in this review. Therefore, when more than one dose is needed for complete primary immunization (e.g., for infants, children and immunocompromized individuals), vaccine exchange should be avoided and the program should be completed with the vaccine used for the first injection.

## List of Abbreviations

ADH: adipic acid di-hydrazide
BCG: Bacillus Calmette-Guerin
CDAP: 1-cyano-4-dimethylaminopyridinium tetrafluoroborate
CRM_197_: Cross Reacting Material 197
DT: diphtheria toxoid
DTaP: diphtheria-tetanus-acellular pertussis
DTwP: diphtheria-tetanus-whole-cell pertussis
ELISA: enzyme-linked immunosorbent assay
GMT: geometric mean titer
Hib: *Haemophilus influenzae* type b
HPV: human papillomavirus
IgG: immunoglobulin G
IMD: invasive meningococcal disease
IPV: inactivated poliovirus vaccine
IU: international unit
Lf: limit of flocculation
Men: meningococcal serogroup
LOS: lipooligosaccharide
Nm: *Neisseria meningitidis*
OAc+: O-acetylated polysaccharide
OAc-: de-O-acetylated polysaccharide
OMPC: outer membrane protein complex
PBS: phosphate buffered saline
PD: protein D
PEG: polyethylene glycol
ppm: parts-per-million
PRP: polyribosyl-ribitol phosphate
SBA: serum bactericidal antibody
SDS-PAGE: sodium dodecyl sulfate-polyacrylamide gel electrophoresis
SEC-HPLC: size-exclusion chromatography-high performance liquid chromatography
SmPC: summary of product characteristics
TdaP: tetanus-diphtheria-acellular pertussis
Th1: type 1 T helper cell
Th2: type 2 T helper cell
Tris: tris(hydroxymethyl)-aminomethane
TT: tetanus toxoid
UK: United Kingdom
WHO: World Health Organization

## List of abbreviations for individual vaccines

HibMenC-TT: *Menitorix*^™^
HibMenCY-TT: *MenHibrix*^™^
MenA-TT: *MenAfriVac*
MenACWY-CRM: *Menveo*^™^
MenACWY-DT: *Menactra*
MenACWY-TT: *Nimenrix*
MenC-TT: *NeisVac-C*
Menin-CRM: *Meningitec*
Menju-CRM: *Menjugate*^™^

## Trademarks

*Bexsero*, *MenHibrix, Menitorix, Menjugate*, *Menveo* and *Synflorix* are trademarks of the GSK group of companies.

*Fluad* is a trademark of Seqirus.

*Menactra* and *TetraMen-T* are trademarks of Sanofi Pasteur.

*MenAfriVac* is a trademark of Serum Institute of India Ltd.

*MeningACon* is a trademark of BeijingZhifei Lvzhu Biopharmaceutical Co., Ltd.

*Meningitec* is a trademark of of Nuron Biotech.

*NeisVac-C* is a trademark of Pfizer.

*Nimenrix* is a trademark of the GSK group of companies, licensed to Pfizer.
